# Hyperbilirubinemia Protects against Aging-Associated Inflammation and Metabolic Deterioration

**DOI:** 10.1155/2016/6190609

**Published:** 2016-07-31

**Authors:** Jaroslav Zelenka, Aleš Dvořák, Lukáš Alán, Marie Zadinová, Martin Haluzík, Libor Vítek

**Affiliations:** ^1^Department of Biochemistry and Microbiology, University of Chemistry and Technology Prague, Technická 5, 166 28 Prague, Czech Republic; ^2^Institute of Physiology, Czech Academy of Sciences, Vídeňská 1083, 142 20 Prague, Czech Republic; ^3^Institute of Medical Biochemistry and Laboratory Diagnostics, 1st Faculty of Medicine, Charles University in Prague, Na Bojišti 3, 128 08 Prague, Czech Republic; ^4^Institute of Biophysics, 1st Faculty of Medicine, Charles University in Prague, Salmovská 1, 128 08 Prague, Czech Republic; ^5^Institute of Endocrinology, Národní 8, 116 94 Prague, Czech Republic; ^6^4th Department of Internal Medicine, 1st Faculty of Medicine, Charles University in Prague, U Nemocnice 2, 128 08 Prague, Czech Republic

## Abstract

Mild constitutive hyperbilirubinemia is associated with a reduced risk of cardiovascular diseases, diabetes, and cancer. Since these pathologies are associated with aging, inflammation, and oxidative stress, we investigated whether hyperbilirubinemia interferes with ROS homeostasis in cell cultures and with inflammation, senescence, and mitochondrial dysfunction in aged rats. Human embryonic kidney cells and rat primary fibroblasts showed a dose-dependent decrease in the ratio of oxidized/reduced glutathione, intracellular H_2_O_2_ levels, and mitochondrial ROS production, with increasing bilirubin concentrations in the culture media. Compared to their normobilirubinemic siblings, aged hyperbilirubinemic Gunn rats showed significantly smaller amounts of visceral fat, better glucose tolerance, and decreased serum levels of proinflammatory cytokines TNF*α*, IL-1*β*, and IL-18. Simultaneously, livers from Gunn rats showed decreased expression of senescence markers and cell cycle inhibitors p21 and p16. Mitochondria from aged Gunn rats showed higher respiration and lower H_2_O_2_ production compared to controls. In conclusion, we demonstrated that mildly elevated serum bilirubin is generally associated with attenuation of oxidative stress and with better anthropometric parameters, decreased inflammatory status, increased glucose tolerance, fewer signs of cellular senescence, and enhanced mitochondrial function in aged rats.

## 1. Introduction

Bilirubin is a natural metabolic end-product of heme breakdown. While its extreme accumulation in neonatal brain under specific pathologic conditions is responsible for oxidative stress and neurotoxicity [1], bilirubin has been increasingly recognized as a potent endogenous antioxidant [2, 3] when only mildly elevated.* In vitro* studies have shown that it may accumulate in cellular membranes [4] and is capable of scavenging reactive nitrogen species [5] as well as peroxyl- and hydroxyl radicals [2, 3], inhibiting lipid peroxidation [3, 6], or even reacting with superoxide and hydrogen peroxide [7].

In contrast to numerous other antioxidants [8], protective effects of bilirubin against a wide array of aging-associated pathologic conditions have been proved in numerous recent studies. Indeed, clinical trials have demonstrated that mild unconjugated hyperbilirubinemia (typically present in subjects with Gilbert syndrome) is significantly associated with lower incidence of cardiovascular and pulmonary diseases, diabetes, metabolic syndrome, certain cancers, and even reduced overall mortality [9]. Gilbert syndrome, caused by a partial deficiency of hepatic bilirubin UDP glucuronosyl transferase (UGT1A1), leads to elevation of unconjugated bilirubin from approximately 10 to about 20–70 *μ*M, without any signs of liver damage [1, 9]. In addition, animal studies employing congenitally hyperbilirubinemic Gunn rats with a complete deficiency of UGT1A1 showed that hyperbilirubinemia has potent anti-inflammatory [10, 11], antiproliferative [12, 13], antigenotoxic [14], antihypertensive [15, 16], and blood lipid-modulating properties [17, 18].

Aging and associated pathologies including cardiovascular diseases, diabetes, and cancer represent major global health problems [19]. Aging is marked by a rise of oxidative damage [20], chronic inflammation [21], development of insulin resistance [22], increased occurrence of senescent cells [23], and mitochondrial dysfunction [24]. It has been shown that these phenomena directly contribute to development of aging-associated pathologies [20–24].

We have previously demonstrated that bilirubin-related tetrapyrroles from blue-green alga* Spirulina platensis* possess strong antiproliferative effects in pancreatic cancer cells through decrease in mitochondrial superoxide levels and consequent shift to reduction in glutathione redox buffer [25]. Here, we aimed at investigating whether hyperbilirubinemia observed in Gilbert subjects and Gunn rats could also alter the ROS homeostasis and whether this effect could lead to attenuation of inflammation, senescence, and mitochondrial dysfunction associated with aging thus protecting against the aging-associated pathologies.

## 2. Materials and Methods

### 2.1. Animals

Hyperbilirubinemic Gunn rats (RHAjj) and their normobilirubinemic siblings (RHAJj) bred in the Centre of Experimental Biomodels, 1st Faculty of Medicine, Charles University in Prague and fed standard chow were grown until age 12–18 months under the supervision of an experienced animal caregiver (MZ) who analyzed their lifelong behavior and feeding characteristics. At the end of the study, the animals were anesthetized with ketamine/xylazine, and 1 mL of blood was collected from the orbital sinus of the left eye. Next, they received 0.2 mL of 30% glucose solution into the tail vein. After 10 minutes, another 1 mL of blood was withdrawn from the orbital sinus of the right eye. After 60 minutes, 5 mL of blood was withdrawn from the vena cava. Their livers, visceral fat, and gastrocnemius muscles were quickly excised and snap-frozen in liquid nitrogen. A tissue graft for establishment of primary culture was taken from the external ear. The local animal research committee approved the protocols for all aspects of the animal studies in accordance with the Guide for the Care and Use of Laboratory Animals, as adopted and promulgated by the United States National Institute of Health.

### 2.2. Preparation of Liver Mitochondria

Liver tissue was homogenized in 180 mM KCl, 5 mM potassium-MOPS, and 5 mM potassium-EGTA (pH 7.2) with a Potter-Elvehjem tissue grinder, and the mitochondria were isolated by differential centrifugation as described previously [26].

### 2.3. Serum Biochemistry and Cytokine Analyses

Serum glucose and bilirubin were analyzed with an automatic analyzer (Modular, Roche Diagnostics GmbH, Basel, Switzerland), using standard laboratory assays. Inflammatory cytokines (leptin, IL-1*β*, IL-10, IL-18, and TNF-*α*) were measured by Luminex technology (using a multiplex kit from EMD Millipore, Darmstadt, Germany).

### 2.4. Cell Culture

Rat primary fibroblasts excised from the ears of euthanized normobilirubinemic animals (see Section 2.1) and human embryonic kidney T-Rex-293 cells (Life Technologies, Carlsbad, CA, USA) were cultured in Dulbecco modified Eagle medium with 5 mM glucose, 3 mM glutamine, and 10% fetal bovine serum, in an atmosphere containing 5% CO_2_. Media enriched with bilirubin (Sigma-Aldrich, St. Louis, MO, USA; purified according to [27]) were prepared from serum directly mixed with a stock solution of 5 mM bilirubin in 0.1 M NaOH. Rat primary fibroblasts were seeded at a constant density, and the rate of proliferation was monitored in every passage. T-Rex-293 cells were transfected with pHyPer-cyto (for detection of cytoplasmic production of H_2_O_2_) and pHyPer-dMito plasmids (for detection of mitochondrial production of H_2_O_2_) (Evrogen, Moscow, Russia), encoding protein H_2_O_2_ sensor HyPer [28], using Lipofectamine 2000 transfection reagent (Life Technologies).

### 2.5. Cell Culture Functional Studies

The proportions of senescent fibroblasts were estimated as the percent of positive cells after staining for senescence-associated *β*-galactosidase using X-Gal (Sigma) substrate at pH 6.2. The samples were inspected with an inverted microscope as described in [29]. WST-1 assay for cell viability was performed according to manufacturer protocol (Roche). Cell proliferation was assessed as changes in the total protein content using a Bradford method (Sigma).

### 2.6. Western Blots

The phosphorylation of S6 protein, a marker of mTORC1 activity, in fibroblast total cell lysate was determined as the ratio between phospho-S6 and total S6 signal on a western blot. The stabilization and nuclear translocation of Nrf2 protein in T-Rex-293 cells were determined by western blot from nuclear lysates as described previously [30]. The antibodies were from Cell Signaling Technology (Danvers, MA, USA) and used according to the manufacturer's protocol.

### 2.7. Gene Expression and Mitochondrial DNA Copy Number

Total liver and fibroblast DNA was isolated with solvent extraction as described elsewhere [31]. Total liver RNA was isolated with Trizol reagent (Life Technologies) according to the manufacturer's protocol and subjected to cDNA preparation with maxima reverse transcriptase (Thermo Scientific, Waltham, MA, USA) using oligo dT primers. Mitochondrial DNA copy number per cell, a marker of mitochondrial abundance, was determined with real time PCR as the ratio of the abundance of a template coded with mitochondrial DNA and a template coded with nuclear DNA as described previously [32]. Expressions of mRNA for p21 and p16, cell cycle inhibitors, which serve as markers of senescent cells [23], as well as GAPDH (used as a reference), were determined with qRT-PCR with following primers:

 p21fwd–TATGTACCAGCCACAGGCAC.
 p21rev–CACGCTCCCAGACGTAGTTG.
 p16fwd–GATTCGAACTGCGAGGACCC.
 p16rev–GTTATGCCTGTCGGTGACCC.
 GAPDHfwd–ATGTCAGATCCACAACGGATACA.
 GAPDHrev–AACTCCCTCAAGATTGTCAGCAA.

### 2.8. Production of H_2_O_2_ from Cells and Mitochondria

The production of H_2_O_2_ from intact fibroblasts in PBS containing 5 mM glucose and 3 mM glutamine, or from isolated mitochondria in the isolation buffer with 10 mM glutamate, 2 mM malate, and 10 mM succinate, was determined using the oxidation of the fluorogenic indicator Amplex red (Life Technologies) in the presence of horseradish peroxidase (HRP, Sigma) according to [26, 33].

### 2.9. FACS Analysis of Intracellular ROS

The relative cytoplasmic and mitochondrial levels of H_2_O_2_ were determined in live T-Rex-293 cells, stably expressing HyPer fluorescent protein probe [28], using FACS analysis (BD LSRII, BD Biosciences, CA, USA). HyPer probe has two excitation maxima recorded at 405 nm (empty) and 488 nm (with bound H_2_O_2_) and an emission maximum at 516 nm. Relative levels of H_2_O_2_ were estimated from the 488/405 fluorescence intensity. The production of superoxide in the mitochondrial matrix was determined as the slope of a time-dependent increase in the fluorescence of a selective MitoSOX probe (Life Technologies) in live T-Rex-293 cells and fibroblasts, treated with/without 10 *μ*M rotenone by FACS analysis as described previously [25].

### 2.10. Determination of GSSG/GSH

To estimate the changes in the global cellular redox state, the ratio of oxidized/total glutathione (GSSG/GSH + GSSG) was determined from cell lysates by capillary electrophoresis (Agilent 7100, Agilent, Santa Clara, CA, USA), equipped with polyimide coating with a fused silica capillary (68 cm × 50 *μ*m) as described previously [25, 33].

### 2.11. Analyses of Metabolites

Levels of lactate in the cell culture media and levels of 2-hydroxyglutarate and 2-oxoglutarate in cell lysates were determined with gas chromatography-mass spectrometry (GC 6890N, MD 5973, Agilent Technologies) as described in [33]. Retention times of selected metabolites were confirmed with commercial standards, and detectable specific mass ions were chosen according to their mass spectra. Total amounts of metabolites (expressed as ng per 10^6^ cells) were calculated from the ratio between the respective ions and internal standard ion (*m*/*z* 190) after alignment with standard curves prepared from commercial standards (Sigma).

### 2.12. Mitochondrial Physiology

The respiration of isolated liver mitochondria in the isolation buffer with 10 mM glutamate, 2 mM malate, and 10 mM succinate and that of T-Rex-293 cell and rat fibroblasts in complete media were measured with an Oroboros O2k oxygraph (Oroboros Instruments, Innsbruck, Austria) as described previously [25, 26, 33]. The relative mitochondrial membrane potential in live T-Rex-293 cells treated with B0–B100 media for 24 hours was determined with tetramethylrhodamine (Life Technologies), according to the manufacturer's protocol using spectrofluorimetry (RF-5301-PC, Shimadzu, Kyoto, Japan).

### 2.13. Statistical Analysis

Statistical analyses were performed and graphs created with SigmaPlot software (Systat software, San Jose, CA, USA) and MS Excel (Microsoft, Redmond, WA, USA), respectively. Data are expressed as mean ± SD. The statistical significance of the results were tested with the Student *t*-test (for normally distributed data), Mann-Whitney Rank Sum test (for skewed data), or ANOVA (for multiple comparisons). Differences were considered statistically significant when *p* values were less than 0.05.

## 3. Results and Discussion

### 3.1. Hyperbilirubinemia Decreases Intracellular ROS Levels

**Figure 1:**
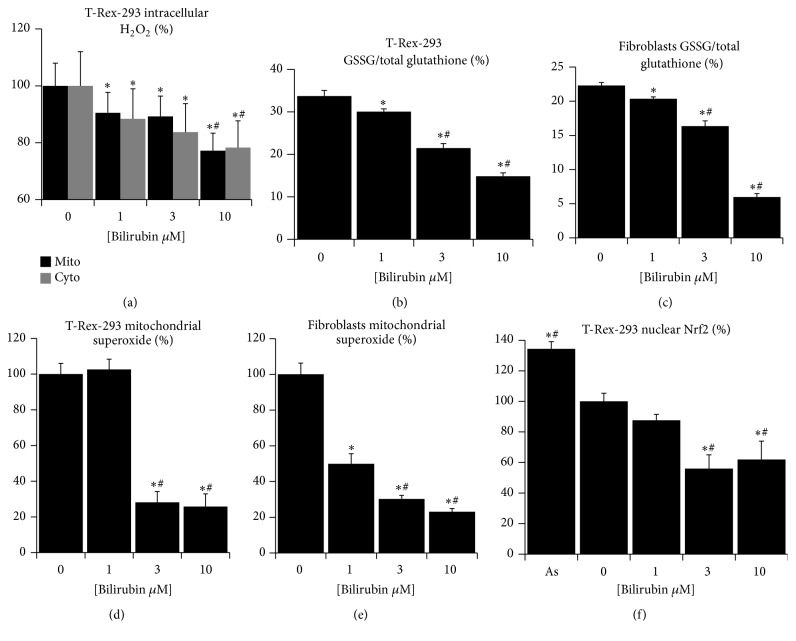
Bilirubin decreases ROS levels in cultured cells. (a) Cytoplasmic (Cyto) and mitochondrial (Mito) levels of H_2_O_2_ in T-Rex-293 cells treated with bilirubin for 24 hours, measured with HyPer protein sensor; (b) ratio of oxidized/total glutathione in T-Rex-293 cells treated with bilirubin for 24 hours, measured with capillary electrophoresis; (c) ratio of oxidized/total glutathione in rat fibroblasts treated with bilirubin for 24 hours, measured with capillary electrophoresis; (d) relative levels of superoxide in mitochondrial matrix measured in T-Rex-293 cells treated with bilirubin for 24 hours, measured with MitoSOX probe; (e) relative levels of superoxide in mitochondrial matrix measured in rat fibroblast treated with bilirubin for 24 hours, measured with MitoSOX probe; (f) nuclear localization of transcription factor Nrf2 in T-Rex-293 cells treated with bilirubin for 24 hours, measured with western blot. Data in panels (a, d, e, and f) expressed as % of controls; *n* = 3 in each group; overall significance measured with ANOVA, *p* < 0.001 in each panel; pairwise comparison with Holm-Sidak method, ^*∗*^significantly different from 0 *μ*M, and ^#^significantly different from 1 *μ*M, *p* < 0.05.

The intracellular ROS are not only damaging at high levels, but their physiological concentrations serve an important cellular signaling mechanisms which influence cell proliferation, metabolism, and function [8, 20, 24]. We have previously demonstrated that phycocyanobilin, a natural tetrapyrrolic compound similar to bilirubin, can attenuate the levels of mitochondrial superoxide and consequently shift the glutathione redox buffer towards reduction [25]. Here, we sought for a similar effect caused by bilirubin under conditions of mild hyperbilirubinemia present in subjects with Gilbert syndrome. Since bilirubin is poorly soluble in water and the vast majority of bilirubin in blood is bound to serum albumin, its biological effects are related to unbound bilirubin (Bf) rather than total bilirubin [1]. Therefore we have treated cells of human (human kidney embryonic T-Rex-293) and rat (primary fibroblasts) origin with culture media containing varying amounts of bilirubin dissolved in 10% fetal calf serum. Following design was used to assess the effect of bilirubin on ROS production: medium with no bilirubin added (NO), medium with 1 *μ*M bilirubin (Bf corresponding to physiological human serum bilirubin concentrations of about 10 *μ*M-NORM), medium with 3 *μ*M bilirubin (Bf corresponding to ~30 *μ*M serum bilirubin concentrations seen in majority of Gilbert subjects, GS), and 10 *μ*M bilirubin (Bf corresponding to ~70 *μ*M, which is the upper limit of bilirubin concentrations in Gilbert syndrome subjects, as well as the bilirubin concentration present in our Gunn rat strain). We found that bilirubin dose-dependently decreased cytoplasmic and mitochondrial levels of hydrogen peroxide (Figure 1(a)) in T-Rex-293 cells expressing HyPer sensor. Moreover, the same treatment decreased the ratio of oxidized/total glutathione in both cell lines in a similar dose-dependent manner (Figures 1(b) and 1(c)). Since mitochondria are considered a principal site of intracellular ROS production [20, 24], we tested whether bilirubin might be able to decrease the amount of superoxide in the mitochondrial matrix after stimulation of its production with rotenone. Indeed, the rate of MitoSOX oxidation was dramatically decreased with increasing concentrations of bilirubin (Figures 1(d) and 1(e)). Recently, Qaisiya et al. demonstrated that bilirubin could activate cellular antioxidant response by stabilization and nuclear translocation of transcription factor Nrf2 [30]. We tested whether this effect could explain the ROS-diminishing effect of bilirubin in our model. In contrast to the observation of Qaisiya et al., we found that nuclear levels of Nrf2 decrease dose-dependently with increasing bilirubin in our cell model (Figure 1(f)), most likely due to much lower and nontoxic bilirubin levels (and Bf) in our experimental setting.

**Figure 2:**
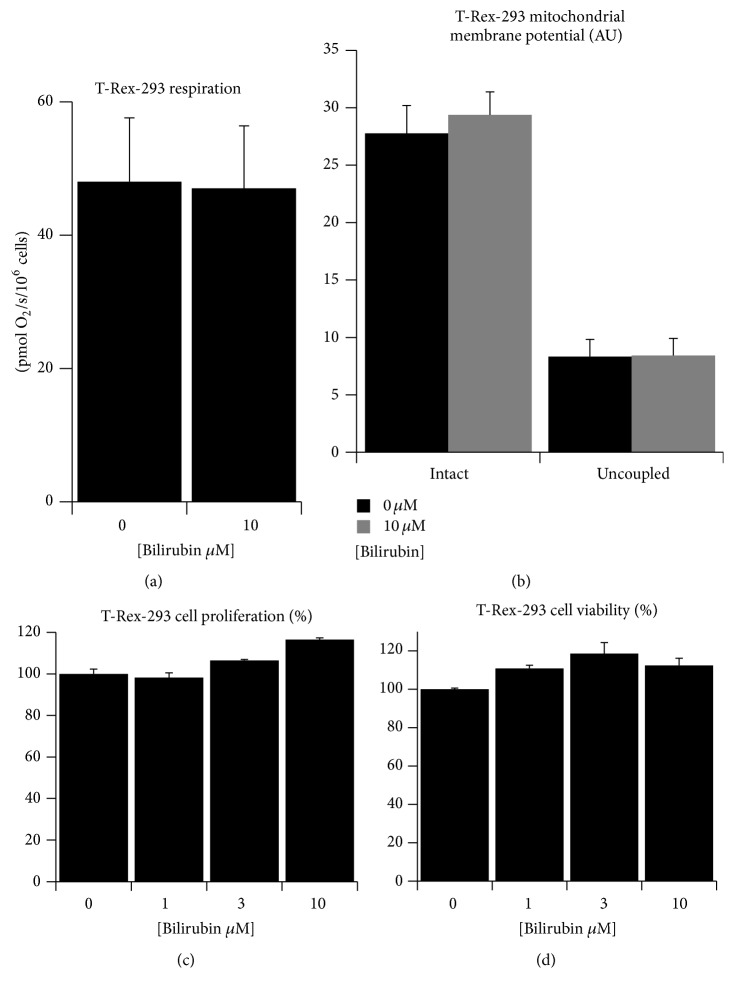
Bilirubin has no negative effect on mitochondria or viability of cultured cells. (a) Whole cell respiration of T-Rex-293 cells treated with bilirubin for 24 hours, measured with respirometry; (b) mitochondrial membrane potential in T-Rex-293 cells treated with bilirubin for 24 hours, measured with tetramethylrhodamine dye. Uncoupling of mitochondria with 10 *μ*M FCCP was performed as a positive control; (c) relative proliferation of T-Rex-293 cells treated with bilirubin for 72 hours, measured as a total cell protein; (d) relative viability of T-Rex-293 cells treated with bilirubin for 24 hours, measured with WST-1 assay. Data in panels (c and d) expressed as % of controls; *n* = 3 in each group; significance in panel (a and b) measured with *t*-test and overall significance in panel (c and d) measured with ANOVA.

In addition, previous reports suggested that bilirubin could also interfere with mitochondrial bioenergetic function [34, 35]. Here, we observed no differences in cell respiration in neither of our experimental media (Figure 2(a)). Moreover, mitochondrial membrane potential measured in the whole cells with tetramethylrhodamine also showed no difference (Figure 2(b)). This lack of inhibition could be explained again by the lower Bf in our experimental setting. Finally, we checked whether the changes in ROS homeostasis could influence viability and proliferation of cells. However, we found no effect of bilirubin on either cell proliferation (Figure 2(c)) or viability (Figure 2(d)).

Thus, we have demonstrated that bilirubin, in concentrations commonly seen in Gilbert subjects and also in our Gunn rats, is dramatically decreasing the mitochondrial levels of superoxide and altering the intracellular ROS homeostasis without compromising mitochondrial function and cell viability. Interestingly, bilirubin has repeatedly been reported to attenuate superoxide production from NADPH oxidases, thus protecting against hypertension [16] and inflammation [11]. However, recent reports suggest that the activity of NADPH oxidases is stimulated by ROS released from the mitochondrial respiratory chain [36]. Therefore, attenuation of NADPH oxidase activity after bilirubin treatment might be interlinked with its inhibitory effect on mitochondrial ROS production observed in our study.

### 3.2. Aged Hyperbilirubinemic Animals Have Favorable Inflammatory and Metabolic Status

**Figure 3:**
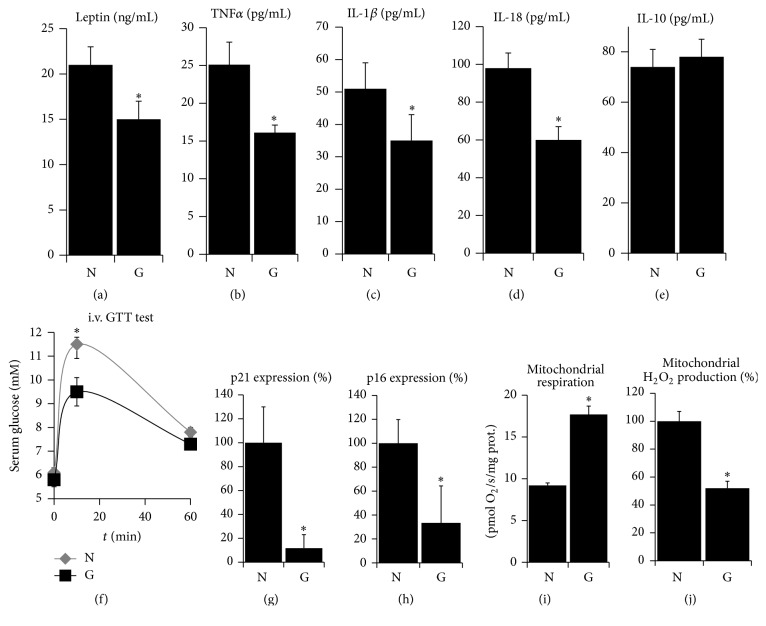
Hyperbilirubinemia protects from inflammation, senescence, and mitochondrial dysfunction in aged rats. Hyperbilirubinemic Gunn rats and their normobilirubinemic siblings aged 12–18 months were evaluated for serum levels of (a) leptin, (b) tumor necrosis factor *α*, (c) interleukin 1*β*, (d) interleukin 18, and (e) interleukin 10; (f) intravenous glucose tolerance test; hepatic mRNA expressions of (g) p21 and (h) p16; and their isolated liver mitochondria were subjected to measurement of (i) respiration and (j) production of H_2_O_2_. N = normobilirubinemic rats and G = Gunn rats; *n* = 5–10 in each group for each measurement; pairwise comparison with *t*-test or Mann-Whitney rank sum test; ^*∗*^significantly different from N; *p* < 0.05.

We hypothesized that a physiological impact of the bilirubin-related changes in ROS homeostasis should be clearly observed in aged individuals due to a longer time of action. Moreover, hyperbilirubinemia is protective against many age-associated diseases [9]. Therefore, we employed aged hyperbilirubinemic Gunn rats to test whether hyperbilirubinemia could influence inflammation, senescence, and mitochondrial dysfunction, the phenomena associated with aging and oxidative signaling [21–24]. Gunn rats represent a well described animal model (and practically the only one for long-term studies) to study not only the toxic effects of hyperbilirubinemia in newborns [1, 37], but also the protective effects of hyperbilirubinemia in adults [1, 6, 11, 13, 15]. Homozygous Gunn rats develop unconjugated hyperbilirubinemia shortly after delivery and maintain serum bilirubin levels close to the upper limit of Gilbert subjects (Table 1) and significantly increased tissue bilirubin levels for their entire lives [37, 38]. In contrast, their heterozygous siblings experience only mild short period of neonatal jaundice followed by bilirubin normalization for the rest of their lives (Table 1) [37]. Although newborn homozygous Gunn rats suffer from severe neonatal jaundice and the consequent cerebellar hypoplasia [37], we observed in this study that the adult animals showed comparable levels of overall activity and the amount of feed ingested (Table 1) as did their normobilirubinemic siblings, which served as controls. The total body weight and the weight of the gastrocnemius muscle were comparable between the Gunn and normobilirubinemic groups (Table 1), suggesting that their nutritional status and overall constitution were similar. However, the weight of a visceral body fat, which is associated with proinflammatory effects and increased mortality [39], was significantly lower in Gunn compared to normobilirubinemic rats (Table 1). This difference correlated well with the lower serum levels of leptin in the Gunn rats (Figure 3(a)). One of the most significant causes of pathologies associated with aging is a systemic low-grade chronic inflammation [21], manifested by elevated serum levels of proinflammatory cytokines and disturbed glucose homeostasis [21, 22]. The onset of chronic inflammation is driven by activation of specific caspases (inflammasomes) which are in turn activated by mitochondrial ROS [40]. Here, we found that serum levels of proinflammatory cytokines TNF*α* (Figure 3(b)), interleukin-1*β* (Figure 3(c)), and interleukin-18 (Figure 3(d)) were lower in aged Gunn rats compared to controls while the level of anti-inflammatory cytokine interleukin-10 was unchanged (Figure 3(e)). The intravenous glucose tolerance test showed better glucose tolerance in aged Gunn rats compared to their normobilirubinemic siblings (Figure 3(f)). The development of chronic inflammation and impaired glucose tolerance in aging is related to the replicative exhaustion of stem cells, leading to the onset of senescence associated with the secretion of proinflammatory cytokines and ROS [23, 41]. Since the onset of senescence is accelerated by elevated mitochondrial ROS [42], we measured the relative mRNA expression of cell cycle inhibitors* p21* and* p16*, generally recognized as markers of senescence [23]. We found that hepatic mRNA expressions of* p21* (Figure 3(g)) and* p16* (Figure 3(h)) were 10 and 3 times lower in aged Gunn rats compared to their normobilirubinemic siblings, respectively. Furthermore, aging is associated with a decline in mitochondrial quality, which results in bioenergetic failure and could be associated with an excessive release of ROS from the dysfunctional respiratory complexes which further enhance the oxidative damage and inflammation of aging [20, 24]. Such mitochondrial dysfunction is presumably caused by a lifelong oxidative damage to mitochondrial proteins and DNA [20, 24, 43]. We assessed the bioenergetics of isolated liver mitochondria from aged Gunn rats and found a higher rate of respiration (Figure 3(i)) and relatively lower production of hydrogen peroxide (Figure 3(j)) compared to mitochondria from normobilirubinemic rats.

Taken together, we found that, in aged animals, hyperbilirubinemia is associated with attenuated chronic inflammation, senescence, and mitochondrial dysfunction. This phenomenon could significantly contribute to prevention of aging-associated pathologies. It could be, therefore, hypothesized that the lower prevalence of cardiovascular diseases, diabetes, and cancer and the lower mortality in Gilbert subjects are at least partially due to their resistance to inflammation and metabolic deterioration associated with aging. 

### 3.3. Bilirubin Attenuates Senescence and Mitochondrial Dysfunction in Primary Fibroblasts

**Figure 4:**
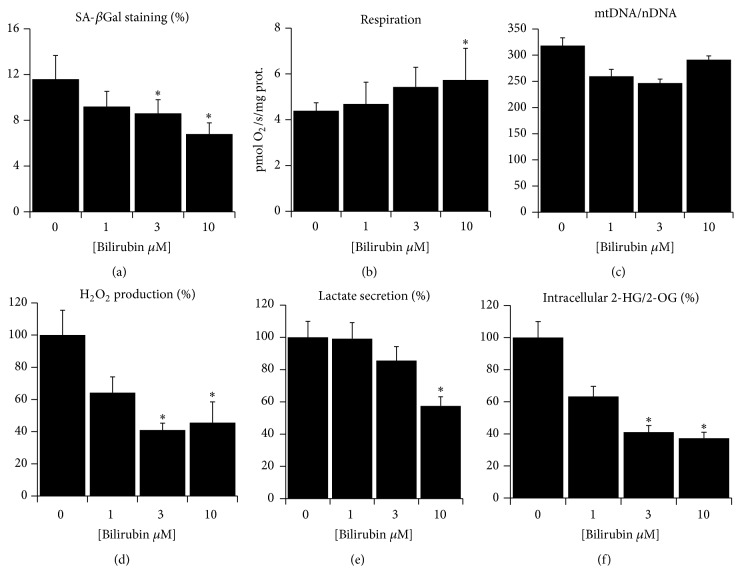
Bilirubin suppresses senescence and mitochondrial dysfunction in cultured primary fibroblasts. Rat primary fibroblasts were cultured for 18 passages in complete media with added bilirubin and then assayed for (a) senescence-associated *β*-galactosidase staining identified with inverted microscope; (b) whole cell respiration measured with respirometry; (c) mitochondrial DNA copy number per cell as a marker of mitochondrial abundance; (d) cellular production of H_2_O_2_ as a marker of ROS homeostasis; (e) lactate secretion to media as a marker of mitochondrial dysfunction; (f) intracellular ratio of 2-hydroxyglutarate/2-oxoglutarate as a marker of mitochondrial dysfunction. Data in panels (a, d, e, and f) expressed as % of controls, *n* = 4–8, overall significance measured with ANOVA, and *p* < 0.05 in each panel; pairwise comparison with *t*-test or Mann-Whitney rank sum test; ^*∗*^significantly different from 0 *μ*M; *p* < 0.05.

Replicative aging of primary fibroblasts in culture is well recognized* in vitro* model to study mechanisms of aging. We have previously validated this model also for studies of mitochondrial dysfunction in aging [33]. Since the Gunn rats are the only animal model suitable for lifelong studies of unconjugated hyperbilirubinemia, we employed replicative aging of fibroblasts as a further support to demonstrate the antiaging properties of bilirubin. Specifically, we tested whether rat primary fibroblasts exposed to higher bilirubin concentrations could be prevented from development of cellular senescence and mitochondrial dysfunction during their replicative aging. We found that high passage fibroblasts (passage >18) exposed to low bilirubin concentrations showed increased positivity for senescence-associated *β*-galactosidase staining, while the opposite phenomenon was observed in cells exposed to higher bilirubin concentrations (Figure 4(a)). In addition, the high passage normobilirubinemic, but not hyperbilirubinemic cells, showed several signs of mitochondrial dysfunction and oxidative stress: the respiration of normobilirubinemic cells was lower (Figure 4(b)) while mitochondrial DNA copy number per cell, a marker of mitochondrial abundance, was similar between all four groups (Figure 4(c)), suggesting that the difference was not due to different level of mitochondrial biogenesis but rather due to differences in the quality of mitochondria. The production of hydrogen peroxide from the aged fibroblast, measured after washing out the bilirubin, was still lower in hyperbilirubinemic cells (Figure 4(d)), suggesting the prevention of excessive ROS release from dysfunctional respiratory chain. The extracellular secretion of lactate and the intracellular ratio of 2-hydroxyglutarate/2-oxoglutarate, both markers of respiratory chain insufficiency [33, 44], was also lower in hyperbilirubinemic cells (Figures 4(e) and 4(f)).

Taken together, the* in vitro* results corroborate the protective effect of bilirubin that we observed in aged hyperbilirubinemic animals and further demonstrate that the protective effect was independent of genotypic or metabolic background of hyperbilirubinemic Gunn rats. Based on our data, we propose that the principal phenomenon underlying the interaction of bilirubin with aging-associated pathologies is the attenuation of mitochondrial ROS levels. Mitochondrial ROS had long been suggested as a cause of aging and negative modulators of lifespan (postulated in the mitochondrial free radical theory of aging) [43], although recent studies focused on manipulation of antioxidant protection provided inconsistent effects on aging and longevity [8]. Nevertheless, some lines of evidence still suggest that the rate of release of ROS into the mitochondrial matrix (not being scavenged with antioxidants) is negatively associated with lifespan and health [20].

## 4. Conclusions

The protective effects of mild elevation of systemic concentrations of bilirubin against aging-associated pathologies have been confirmed in several clinical studies. Here, we demonstrate that clinically relevant, mildly elevated levels of bilirubin potently decrease the mitochondrial and cytoplasmic ROS levels, thus modulating the cellular ROS homeostasis in human and rat cells. This effect is associated with decreased accumulation of visceral fat, lower markers of chronic inflammation, better glucose tolerance, improved status of cellular senescence, and protection against mitochondrial dysfunction in aged hyperbilirubinemic animals. These findings were consistent with protection against cellular senescence and mitochondrial dysfunction observed in cultured primary fibroblasts exposed to increasing bilirubin concentrations in media. Therefore, we suggest that the lower incidence of aging-associated pathologies such as atherosclerosis, cancer, and diabetes in subjects with mild elevation of serum bilirubin might be due to their resistance to aging-associated inflammation and metabolic deterioration, driven by attenuated oxidative signaling.

## Figures and Tables

**Table 1:** Anthropometric and metabolic parameters of 12-month-old hyperbilirubinemic Gunn rats and their normobilirubinemic heterozygous siblings (*n* = 5 in each group).

Parameter	Normobilirubinemic	Gunn	*p* value (*t*-test)
Bilirubin (*μ*M)	1.5 ± 0.4	75 ± 18	^*∗*^0.001
Body weight (g)	309 ± 55	298 ± 33	0.67
Gastrocnemius muscle (g)	2.2 ± 0.2	2.0 ± 0.3	0.22
Visceral fat (g)	6.3 ± 2	3.2 ± 1	^*∗*^0.01
Feed/day (g)	10 ± 2	10 ± 3	0.8

^*∗*^Significant difference between normobilirubinemic and Gunn rats.
